# Relationships between cognitive function and activities of daily living in psychiatric nursing home patients with schizophrenia

**DOI:** 10.1192/j.eurpsy.2024.1578

**Published:** 2024-08-27

**Authors:** F.-Y. Gu, S.-Y. Weng

**Affiliations:** ^1^Department of Occupational Therapy, Taipei City Hospital, Songde Branch; ^2^Taipei City Hospital Songde Branch Psychiatric Nursing Home, Taipei City, Taiwan, Province of China

## Abstract

**Introduction:**

Schizophrenia is a common chronic disease in psychiatric long-term care institutions. Taipei City Hospital Songde Branch Psychiatric Nursing Home (TCHSBPNH) is the first public psychiatric nursing home in Taipei City. There’s not only a complete interdisciplinary care system, but also detail initial evaluation at admission of residents, including basic demographic information, cognitive assessments, activities of daily living, and so on. It is known that patients with schizophrenia are generally accompanied by cognitive impairments, which further affects their activities of daily living (ADL) performance, but we still don’t know the correlation between of them.

**Objectives:**

This study aimed to investigate the relationships between cognitive function and ADL function of psychiatric nursing home residents with schizophrenia. The results would promote clinical intervention in ADL training for institutional patients.

**Methods:**

39 participants with chronic schizophrenia (mean age =63.95±6.59 years) were recruited for the study from 2020 to 2021 in TCHSBPNH. We collected every resident’s assessment data, including Mini-Mental State Examination (MMSE), Barthel Index, Lawton - Brody Instrumental Activities Of Daily Living Scale (Lawton IADL scale) and Composite Physical Function (CPF) Scale. The Pearson correlation coefficient was used to examine the correlation between cognitive function and ADL.

**Results:**

Moderate positive correlations were showed between cognitive function and ADL (*p*<.05). The statistic results as follow, MMSE and Barthel Index (γ=.627, *p*<.001), Lawton IADL scale (γ=.431, *p*=.006), and CPF (γ=.341, *p*=.034) respectively exhibited significant correlations.

**Image:**

**Figure:**
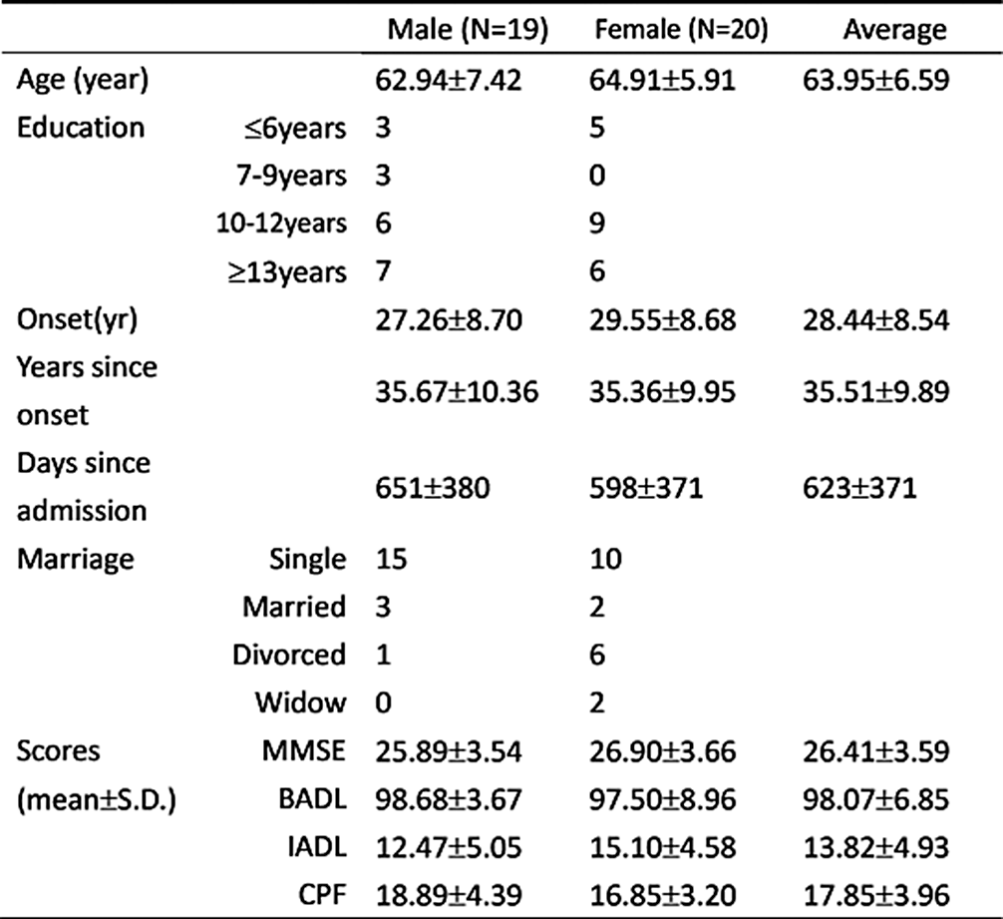

**Image 2:**

**Figure:**
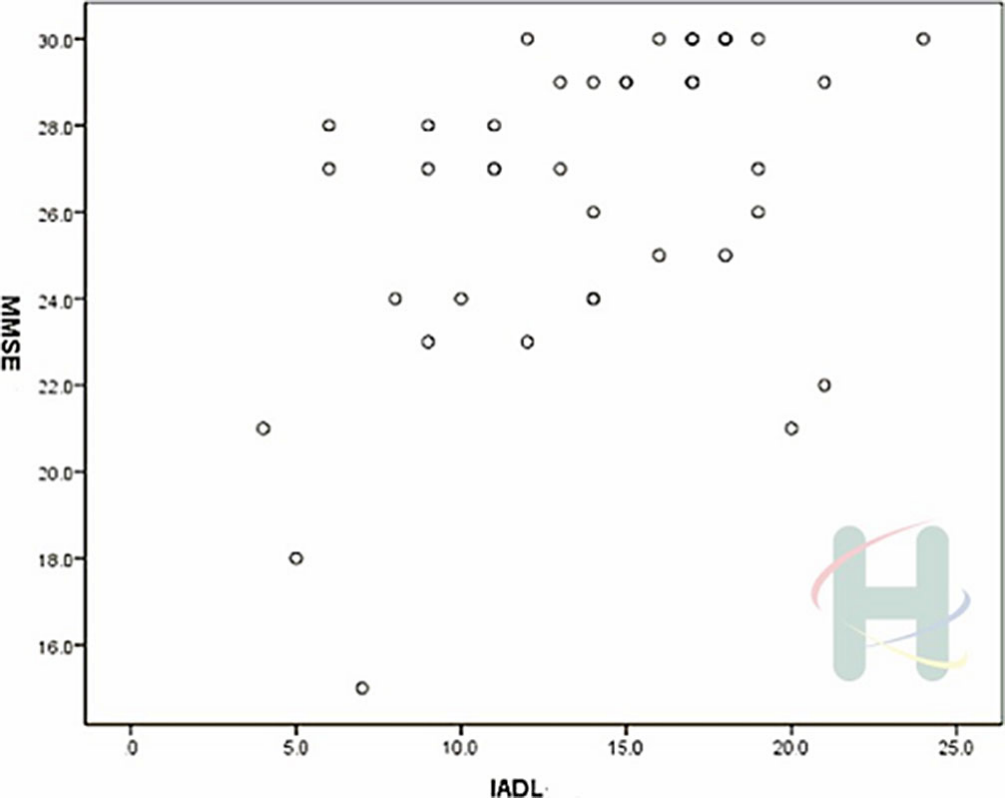

**Image 3:**

**Figure:**
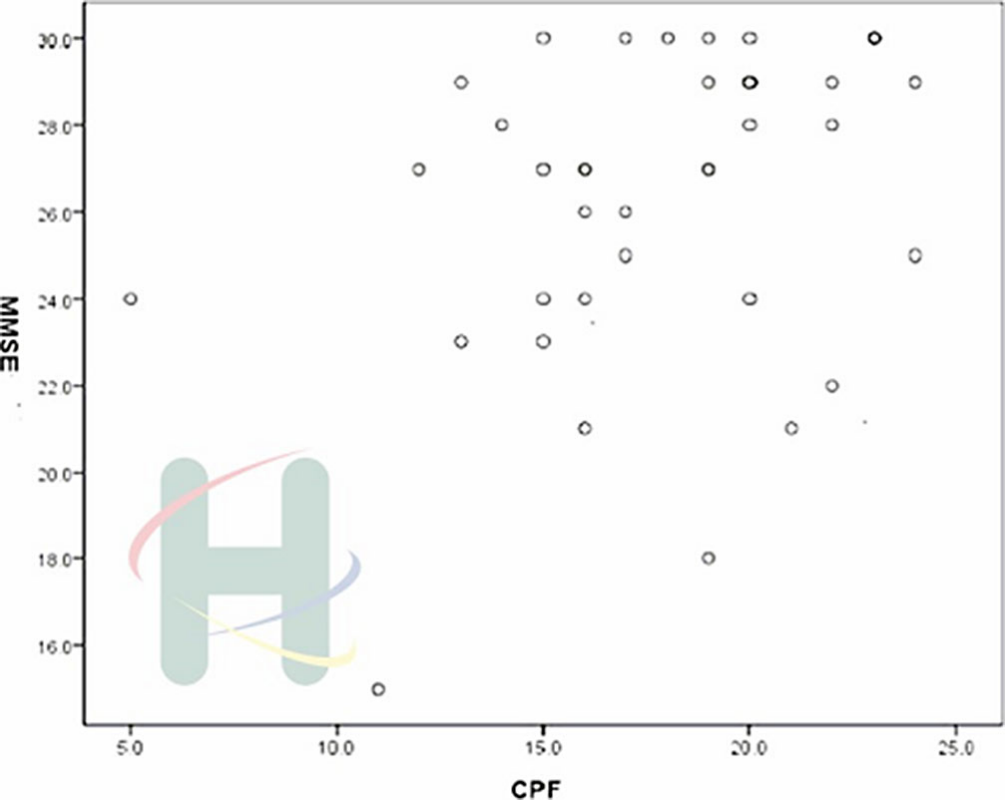

**Conclusions:**

There is a positive correlation between cognitive function and ADL in psychiatric nursing home residents with schizophrenia. The better cognitive function performance becomes, the better independent ADL functions will be. Thus, the psychiatric nursing home residents’ independent ADL training will also vary from person to person.

**Disclosure of Interest:**

None Declared

